# Supplementary material for: Enhancing Clinical Reasoning with Virtual Patients: A Hybrid Systematic Review Combining Human Reviewers and ChatGPT
Source: Healthcare (Basel). 2024 Nov 11;12(22):2241. doi: 10.3390/healthcare12222241 (PMC11594149; doi:10.3390/healthcare12222241)
Supplement: Supplementary file 1 [file healthcare-12-02241-s001.zip › healthcare-3240394-supplementary.pdf]

Supplementary Files

Supplementary File S1. Independent Prompt and GPT Responses

Independent Prompt
Acts as an expert reviewer of scientific articles.

Create a table with this 9 columns:
| Article title | Summary | Software/AI used | Software/AI Description | Participants | Control and other groups | Hospital Area | Learning Impact | Student Satisfaction |

I am going to attach a scientific article to you and I need to extract the following information:

1- Article title
2- Summarize the article in less than 200 words.
3- What software, type of software, AI or AI algorithm do you use? Indicate only the answer, if it is not available also indicate it.
4- Brief description of the software or AI used
5- Indicates the number of participants in the study.
6- Indicates if there is a control group. What other groups are there?
7- In which hospital area is it performed: nursing, medicine, or others?
8- What is the result that the use of the virtual patient causes on learning? List it in different points.
9- Is the degree of satisfaction of the students in the study mentioned? If yes, indicate which one it is.

Do not give redundant or unnecessary information.

GPT4 Responses

Article title	Summary	Software/AI used	Participants	Control and other groups	Hospital Area	Learning Impact	Student Satisfaction
The use of a virtual patient case in an OSCE-based exam – A pilot study	This pilot study evaluated the integration of a virtual patient (VP) into an OSCE exam to assess medical students' clinical reasoning and problem-solving skills. 118 fourth-year medical students at Karolinska Institutet participated. The study found that VPs might be useful for assessments in medical education, suggesting they can reliably differentiate student performance and provide a realistic and engaging testing environment. However, the study also recognized the influence of human assistants on student outcomes and emphasized the necessity for proper training and system familiarity among students to effectively use VP systems like ISP for assessments.	ISP system	118	No control group mentioned, other groups not specified	Medicine	- VP can reliably differentiate student performance. - It provides a realistic testing scenario. - Encourages active and reflective learning.	
The use of a virtual patient case in an OSCE-based exam – A pilot study	This study evaluated the use of a virtual patient (VP) simulation tool, the ISP system, in an OSCE-based clinical reasoning and problem-solving assessment among 118 fourth-year medical students at Karolinska Institutet. The VP aimed to test clinical skills in a realistic yet controlled environment, enhancing learning through interactive simulation. The results highlighted the ISP's potential in differentiating student performances effectively, although adjustments for assistant influence and student training on the system were recommended.	Interactive Simulation of Patients (ISP)	118 fourth-year medical students	No specific control group mentioned, but students were divided into subgroups across four hospitals.	Medical education, specifically within a surgical course at Karolinska Institutet	The ISP system helped in assessing clinical reasoning and problem-solving skills effectively. Key impacts included: - Differentiating student performance reliably - Highlighting the need for student familiarization with the system - Suggesting potential for high-stakes exams with further development	Student satisfaction varied, with a general positive response towards the engaging and realistic nature of the simulation. Some students highlighted concerns about the system's interface and the limited interactive dialogue with the virtual patient.
The use of a virtual patient case in an OSCE-based exam – A pilot study	This study evaluated the use of a virtual patient (VP) in an OSCE-based exam among 118 fourth-year medical students at Karolinska Institutet. It aimed to assess VP as a tool for clinical reasoning and problem-solving assessment. The study observed students' interactions with the ISP system for history taking, physical examination, and diagnosing. Results indicated that VPs could differentiate student performance effectively, though some logistical and technical adjustments were necessary.	Interactive Simulation of Patients (ISP)	118 fourth-year medical students	Not explicitly a control group but comparisons were made between different student groups based on the assistance received.	Medicine, within the surgery course context at university hospitals.	VP was effective in differentiating between students' performances and provided a realistic, engaging learning environment. However, improvements are needed in system familiarity and reducing the influence of human assistants.	Mixed results; while students found the VP engaging and useful, some noted limitations in interaction and system unfamiliarity.
Article title	Summary	Software/AI used	Participants	Control and other groups	Hospital Area	Learning Impact	Student Satisfaction
The virtual patient as a learning tool	This study investigates the effectiveness of Virtual Patients (VPs) as low-fidelity simulators for improving clinical reasoning and history taking skills in undergraduate medical students at Universidad del Rosario. The course incorporated VPs in a constructivist educational framework, allowing students to practice and refine essential medical skills without patient risk. Results showed significant improvement in students' skills from pre- to post-course assessments. VPs were well-received, providing a risk-free environment for error, promoting self-directed learning, and facilitating a deeper understanding of medical practices through repetitive, focused practice.	Virtual Patient: Simulator of Clinical Cases (Web-based)	20 medical students	No control group; study group of 20 undergraduate medical students from first to fifth academic periods.	Medicine	Significant improvements in history taking and clinical reasoning skills, confirmed by pre- and post-course evaluations.	High satisfaction, with students finding the VP easy to use, motivating, and beneficial for learning without stress, particularly at the start of their careers.
The virtual patient as a learning tool: a mixed quantitative qualitative study	The study explores the effectiveness of a low-fidelity simulator, Virtual Patient (VP), in developing clinical reasoning and history-taking skills among medical students. The course used VP extensively and showed significant improvements in students' abilities across various clinical skills through a mix of quantitative and qualitative methods.	Virtual Patient (Web-based software)	20 undergraduate medical students	No specific control group; pre-and-post course evaluations were used for all participants.	Medicine	Significant improvements were noted in students' interview skills, physical examination, clinical judgment, relevance of medical exams, and case presentation abilities.	High student satisfaction reported; students found VP easy to use, stress-reducing, and helpful in making mistakes without real-world consequences.
The virtual patient as a learning tool: a mixed quantitative qualitative study	The study evaluated the effectiveness of a Virtual Patient (VP) simulator in teaching clinical reasoning and history-taking skills to undergraduate medical students using a low-fidelity simulation. The VP was used in a constructivist learning environment to improve essential medical skills.	Virtual Patient (Web-based tool)	20 undergraduate medical students	None mentioned	Medicine	1. Enhanced interview skills<br>2. Improved physical exam skills<br>3. Enhanced clinical judgment<br>4. Improved relevance of medical exams<br>5. Better case presentation skills	Yes, students found the VP to be a stress-free and motivating learning tool.
Article title	Summary	Software/AI used	Participants	Control and other groups	Hospital Area	Learning Impact	Student Satisfaction
Learning clinical reasoning with virtual patients	This study evaluates a virtual patient (VP) system integrated with AI and natural language processing, aimed at teaching medical students clinical reasoning. It involves students interacting with a VP simulating medical issues, helping them build clinical reasoning by making diagnoses based on the VP's responses. The system provided feedback and performance scores, enhancing self-directed learning and clinical competence. The methodology was tested on 112 medical students at Kaohsiung Medical University and E-Da Hospital in Taiwan, showing that it effectively enhances students' clinical problem-solving skills and confidence.	AI with natural language processing	112 medical students	Not explicitly mentioned	Medicine	Enhanced clinical reasoning, confidence in handling real patients, and structured thought patterns.	High student satisfaction; 80% reported increased confidence and proficiency, with a preference for more VP-based learning experiences.
Learning clinical reasoning with virtual patients	This study addresses the challenges of teaching clinical reasoning in medical education, traditionally handled through lectures and case studies. With the introduction of advanced technology, a virtual patient (VP) system utilizing artificial intelligence and natural language processing was developed to enhance learning through simulated clinical encounters. The study evaluated the effectiveness of this VP system with 112 medical students at Kaohsiung Medical University and E-Da Hospital, focusing on its impact on students' clinical reasoning skills and confidence.	Virtual patient (VP) system with artificial intelligence and natural language processing	112	Not specified	Medical (Kaohsiung Medical University and E-Da Hospital)	- Early clinical exposure to patient scenarios<br>- Opportunities for repetitive practice and immediate feedback<br>- Enhancement of self-directed learning and knowledge structure improvement<br>- Increased confidence and proficiency in clinical problem-solving among students	- High appreciation for the system's contribution to competence in problem-solving<br>- 80% reported increased confidence and proficiency
Learning clinical reasoning with virtual patients	This study introduces a virtual patient (VP) system enhanced by artificial intelligence and natural language processing to facilitate clinical reasoning in medical students. It simulates clinical encounters, allowing students to interact with a VP, make decisions, and receive immediate feedback. The system was tested with 112 medical students in Taiwan, showing benefits in clinical problem solving and learning effectiveness.	Artificial intelligence and natural language processing	112	No control group specified	Medicine (Kaohsiung Medical University and E-Da Hospital)	- Facilitates self-directed learning. - Enhances competence in clinical problem solving. - Improves knowledge structure and decision-making confidence.	- 80% of students reported increased confidence. - Students expressed a desire for more VP-based experiences.
Article title	Summary	Software/AI used	Participants	Control and other groups	Hospital Area	Learning Impact	Student Satisfaction
Using a Virtual Patient via an Artificial Intelligence Chatbot to Develop Dental Students' Diagnostic Skills	This study evaluates the impact of a virtual patient AI chatbot on dental students' diagnostic skills at the Universidad Europea de Madrid. The AI chatbot, named Julia, simulated a patient with reversible pulpitis, providing a safe and controlled environment for students to practice and improve their diagnostic abilities. The chatbot was accessed via Telegram, allowing flexible student interaction. The study found that the use of the chatbot improved students' diagnostic skills and was well-received, suggesting potential for wider curriculum integration.	Dialogflow®	193 fourth and fifth-year dentistry students	No specific control group; study participants used the chatbot in a descriptive cross-sectional study format.	Dental education	- Enhanced diagnostic competence<br>- Increased exposure to clinical cases<br>- Improved self-evaluation and accountability	High satisfaction reported, with an average rating of 4.36 out of 5, indicating positive reception and perceived usefulness of the AI tool in their learning process.
Using a Virtual Patient via an Artificial Intelligence Chatbot to Develop Dental Students' Diagnostic Skills	The study explores the use of an AI chatbot as a virtual patient (named Julia) to enhance diagnostic skills in dental students. It focused on integrating this technology into the dental curriculum, assessing its effectiveness in improving diagnostic accuracy and student satisfaction through interaction with the virtual patient in a controlled, stress-free environment. The findings suggested overall positive student reception and improved diagnostic competencies, recommending further integration of AI into dental training.	Dialogflow® application	193 dental students from the Universidad Europea de Madrid	No control group; study participants were fourth and fifth year students	Dentistry (specifically dental students' education)	The virtual patient (AI chatbot) effectively enhanced diagnostic skills and allowed students to practice in a risk-free environment, thereby reinforcing their learning experience.	High overall satisfaction with mean ratings around 4.36 on a 5-point scale, indicating positive student feedback on their learning experience with the AI chatbot.
Using a Virtual Patient via an Artificial Intelligence Chatbot to Develop Dental Students' Diagnostic Skills	The study explored the impact of an AI chatbot, simulating a virtual patient, on the diagnostic skills of dental students. It involved 193 students from the Universidad Europea de Madrid, who interacted with the chatbot to improve their diagnostic abilities. The chatbot, designed with Dialogflow, used conversational AI to simulate patient interactions and provide feedback on student diagnoses. The study showed high student satisfaction and suggested integrating similar technologies into dental education to enhance learning.	Dialogflow	193 students from 4th and 5th year dentistry	No specific control group mentioned. Study participants were 4th and 5th year dental students.	Dental Education (dentistry)	- Enhanced diagnostic skills through repetitive practice and feedback. - Improved adaptation to technological advancements in dental education.	- Overall high satisfaction with the AI interaction (mean score of 4.36 out of 5). - Higher satisfaction noted among 5th year students.
Article title	Summary	Software/AI used	Participants	Control and other groups	Hospital Area	Learning Impact	Student Satisfaction

Affect and Cognitive Closure in Students—A Step to Personalised Education of Clinical Assessment in Psychology with the Use of Simulated and Virtual Patients	This study examines how affect and cognitive closure influence the acquisition of clinical interview and reasoning skills among psychology students, utilizing simulated (SP) and virtual patients (VP). With 56 fifth-year clinical psychology students participating, the study explored the effectiveness of these teaching methods. SP was found more beneficial for enhancing interview skills, whereas VP was more effective for clinical reasoning skills. The study aims to tailor educational approaches to individual learning capabilities, improving both the educational process and student outcomes in clinical psychology.	The study used the authors' own SP and VP tools to facilitate and measure learning in clinical psychology settings.	56 fifth-year clinical psychology students participated.	Control groups are not explicitly mentioned, but the study involved both SP and VP groups, suggesting a comparative approach.	Not specified as conducted within a hospital setting; it involved university psychology students.	SP enhanced interview skills effectively. VP was beneficial for most clinical reasoning skills, helping those with low efficacy in fulfilling their need for closure (low EFNC).	Not explicitly mentioned, though the study discusses the effectiveness of teaching methods, implying some level of attention to student experience and satisfaction.
Affect and Cognitive Closure in Students—A Step to Personalised Education of Clinical Assessment in Psychology with the Use of Simulated and Virtual Patients	This study investigates the impact of affect and cognitive closure on learning outcomes among clinical psychology students using simulated and virtual patients. It examined how different levels of need for closure affect the development of clinical interview and reasoning skills. The results indicate that simulated patients were more effective for teaching interview skills, while virtual patients helped in learning clinical reasoning skills, especially for students with lower efficacy in fulfilling their need for closure.	The study used specific software tools developed by the authors for virtual and simulated patient interactions, aimed at teaching and evaluating psychological interview techniques and clinical reasoning.	56 fifth-year clinical psychology students participated.	Participants were divided into groups using simulated patients (SP) and virtual patients (VP).	The study was conducted within the academic setting of the psychology department, not in a hospital area.	The study found simulated patients more effective for interview skills, and virtual patients beneficial for clinical reasoning skills, especially among students with low efficacy in fulfilling their need for closure.	Student satisfaction was not explicitly measured or mentioned in the results.
Affect and Cognitive Closure in Students—A Step to Personalised Education of Clinical Assessment in Psychology with the Use of Simulated and Virtual Patients	The study explored the role of affect and cognitive closure in learning clinical assessment skills using simulated and virtual patients among 56 fifth-year clinical psychology students. The research found that simulated patients were more effective for interview skills while virtual patients were beneficial for clinical reasoning skills. This highlights how affect and cognitive needs influence learning methods, suggesting a personalized approach to teaching clinical skills.	The study utilized the authors' tools for simulated patient (SP) and virtual patient (VP) sessions.	56 fifth-year clinical psychology students	There were two groups: one group trained with simulated patients and the other with virtual patients.	This study was conducted at the University of Social Sciences and Humanities, not a hospital, focusing on psychology education.	Simulated patients improved interview skills. Virtual patients were beneficial for clinical reasoning skills, especially for students with lower efficacy in fulfilling the need for cognitive closure.	Not specifically mentioned; the focus was on learning outcomes rather than satisfaction.

Article title	Summary	Software/AI used	Participants	Control and other groups	Hospital Area	Learning Impact	Student Satisfaction
Transformative Learning through Virtual Patient Simulations: Predicting Critical Student Reflections	This article evaluates the use of the Digital Clinical Experience (DCE) simulation software by master of science in nursing students to promote transformative learning through structured self-reflections. The study employed a within-stage mixed-model design analyzing students' performance based on the amount of dialogue and clinical items discovered during the simulation. Results suggest that critical self-reflection, indicative of transformative learning, correlates positively with the exploration of secondary clinical findings, while focus on primary findings alone had a negative correlation with the depth of reflection.	Shadow Health Digital Clinical Experience (DCE)	130 MSN students	No specific control group; the study is based on the performance and self-reflections of students using the DCE.	Nursing	1. Enhanced critical self-reflection. 2. Positive correlation between the exploration of secondary clinical findings and critical reflection.	Not specifically mentioned, but the study implies a focus on the educational effectiveness rather than direct student satisfaction metrics.
Transformative Learning through Virtual Patient Simulations: Predicting Critical Student Reflections	This study investigates the impact of virtual patient simulations on the critical self-reflections of 130 Master of Science in Nursing (MSN) students using the Digital Clinical Experience (DCE) software. It explores how the depth of clinical findings discovered during simulations influences reflective practice, employing a framework of situated cognition and transformative learning theory. The findings suggest that more in-depth exploration of clinical topics in simulations can enhance the quality of students' reflections, potentially leading to transformative learning experiences.	Digital Clinical Experience (DCE)	130 MSN students	Not specified	Nursing education	The study found that critical self-reflection, indicative of transformative learning, was more likely when students uncovered more secondary clinical items during simulations. This suggests that deeper exploration of patient histories and conditions in simulations can enhance learning outcomes.	Not explicitly mentioned
Transformative Learning through Virtual Patient Simulations: Predicting Critical Student Reflections	This study utilized the Shadow Health Digital Clinical Experience (DCE), a virtual patient simulation, to assess the impact on nursing students' critical self-reflection. It involved MSN students who engaged in virtual patient interactions aimed at enhancing their clinical reasoning and reflective journaling skills. The study's findings suggest that deeper engagement with varied clinical findings during the simulation can promote more critical self-reflections, which is crucial for transformative learning experiences.	Shadow Health Digital Clinical Experience (DCE)	130 MSN students	Not specified	Nursing education	1. Enhanced clinical reasoning skills through interactive simulations. 2. Deeper self-reflection linked to handling complex patient cases.	Not explicitly mentioned
Article title	Summary	Software/AI used	Participants	Control and other groups	Hospital Area	Learning Impact	Student Satisfaction
Hospital nurses' management of agitation in older cognitively impaired patients: do they recognise pain-related agitation?	This study examines how hospital nurses manage agitation in cognitively impaired elderly patients, specifically whether they can identify pain-related causes. Nurses often rely on antipsychotics rather than recognizing and treating the underlying pain. The study highlights a gap in current nursing practice and emphasizes the need for better pain management strategies in hospital settings.	Virtual Human Toolkit	274 nurses	Not applicable	Medical and surgical settings in hospitals	The simulation exposed a significant gap in recognizing and managing pain-related agitation, demonstrating an over-reliance on antipsychotics.	Not explicitly mentioned in the provided details
Hospital nurses' management of agitation in older cognitively impaired patients: do they recognise pain-related agitation?	This study investigates how hospital nurses manage agitation in older cognitively impaired patients, focusing on whether they recognize agitation caused by pain. A virtual simulation was used to assess nurses' responses in managing patient agitation without prior information about the patient's pain condition. The findings revealed that most nurses (95%) did not identify pain as a cause of agitation and predominantly used antipsychotics for management. The study highlighted a significant gap in recognizing pain-related agitation, suggesting a need for improved training and awareness .	Virtual Human Toolkit	274 registered nurses	No specific control group; the study involved various nurse participants undergoing the simulation independently .	Medical and surgical settings in public hospitals	The simulation demonstrated a significant gap in the practice of recognizing pain-related agitation, with only 5% of nurses identifying pain as a source of agitation correctly .	Not explicitly mentioned; however, the simulation's reliability and immersion were rated highly, which could indirectly indicate participant engagement and satisfaction .
Hospital nurses' management of agitation in older cognitively impaired patients: do they recognise pain-related agitation?	This descriptive correlational study investigates how hospital nurses manage agitation in older cognitively impaired patients experiencing pain, using a virtual simulation. It revealed a significant reliance on antipsychotics with inadequate patient assessment, highlighting a practice gap in recognizing and managing pain-related agitation.	Virtual Human Toolkit	274 nurses	No control group; the study was cross-sectional with multivariate descriptive design.	Medical and surgical settings in hospitals	The study illuminated the gap in practice and poor management of pain-related agitation by nurses, which is often mismanaged as behavioral issues rather than pain.	Not explicitly mentioned in the summary provided
Article title	Summary	Software/AI used	Participants	Control and other groups	Hospital Area	Learning Impact	Student Satisfaction
User Response to the Simulation of a Virtual Patient with Cranial Nerve Injury	The study evaluated the effectiveness of the Neurological Examination Rehearsal Virtual Environment (NERVE) in simulating a cranial nerve exam for medical students, residents, and clinicians. It assessed their ability to identify cranial nerve lesions and gauged their satisfaction with the technology. Despite some technical limitations, NERVE was found to have educational value, particularly for residents who benefited from repeated exposure .	NERVE	69	No specific control group mentioned	Not specified	- Improved identification of cranial nerve lesions, especially with repeated exposure.	
User Response to the Simulation of a Virtual Patient with Cranial Nerve Injury	The study evaluated the effectiveness of the Neurological Examination Rehearsal Virtual Environment (NERVE) tool, which is a virtual patient simulation designed to help medical students, residents, and clinicians improve their skills in diagnosing cranial nerve deficits. Despite technical limitations, participants found the tool beneficial for learning. The study involved various metrics to assess learning, satisfaction, and the realism of the simulation.	NERVE	69 participants: 9 clinicians, 7 residents, and 53 medical students	No control group; participant groups include clinicians, residents, and medical students at various levels.	Not specified as a specific hospital area, used broadly for medical education.	NERVE was found to be beneficial in enhancing examination skills, especially for identifying cranial nerve deficits. The tool was particularly useful for residents, who had the lowest initial success in correct diagnosis but benefited from repeated exposure.	The students reported a positive educational experience with NERVE despite some technical limitations. Overall satisfaction with the tool was moderate to high across different groups, with specific praise for its educational value .
Neurological Examination Rehearsal Virtual Environment (NERVE)	The study evaluates the effectiveness of the NERVE tool, a virtual patient simulation developed for cranial nerve examination training. The simulation was used by medical students, residents, and clinicians to improve neurological examination skills. Despite some technical limitations, such as issues with the speech recognition software, participants generally found NERVE beneficial for learning, although satisfaction levels varied with some technical criticisms. The research aims to enhance neurological diagnostic skills through repetitive exposure and improved simulation technology.	NERVE	69 participants (9 clinicians, 7 residents, 53 medical students)	Not specifically mentioned as a control group; the study appears to involve only one group using the NERVE system.	Medical education in neurology	NERVE was positively received for its educational value in improving examination techniques, though technical issues were noted. Benefits included enhanced familiarity with neurological exams and the ability to practice rare clinical scenarios.	Mixed: Overall, participants acknowledged the educational benefits, though they noted technical limitations and varying levels of realism in the simulation.
Article title	Summary	Software/AI used	Participants	Control and other groups	Hospital Area	Learning Impact	Student Satisfaction
Developing a Conversational VSP	This article describes the development of a virtual standardized patient (VSP) system intended to train medical students in history-taking skills. The VSP engages students in natural language conversations, simulating real-life patient interactions. The study tests the system's conversational accuracy and its impact on student learning and diagnostic skills.	ChatScript	52 medical students	No control group specified	Not specified	The system demonstrated a high degree of conversational accuracy with responses deemed accurate between 79% to 86% of the time, depending on the case complexity. Students were able to practice and improve their differential diagnosis skills effectively.	Not specifically mentioned

Developing a Conversational VSP	The article describes the development and implementation of a virtual standardized patient (VSP) system to enhance medical students' history-taking skills. The VSP system utilizes a natural language processing dialogue management system integrated with 3D characters to simulate realistic patient interactions. This allows students to practice clinical reasoning and differential diagnosis development before engaging with real patients. The system's feedback aims to improve students' diagnostic accuracy and communication skills.	ChatScript and Unity 3D	52 students (18 third year, 34 first year)	Control group not specified. Third-year and first-year medical students participated in different complexity scenarios.	Not specified, likely medical education or clinical simulation.	- Improved diagnostic accuracy. - Enhanced clinical reasoning. - Better history-taking skills.	Not explicitly mentioned in the text, but implied positive based on system feedback improvements.
Developing a Conversational Virtual Standardized Patient to Enable Students to Practice History-Taking Skills	This article discusses the development and implementation of a virtual standardized patient (VSP) system, which integrates a dialogue management system (ChatScript) with 3D characters in a high-fidelity game engine (Unity) to simulate patient interactions for medical students. The system, tested with medical students at the Ohio State University College of Medicine, allows students to practice history-taking skills in a controlled environment. The VSP system aims to improve students' diagnostic accuracy and reduce faculty time and resources, enhancing early skill acquisition before real-life patient encounters.	ChatScript	18 third-year and 34 first-year medical students	The study involved a control group of third-year medical students and other groups of first-year medical students for different complexity levels of cases.	Not specified	The use of VSP improved diagnostic accuracy and confidence in history-taking before real patient interactions. It allows for practice in a non-threatening environment, aiding in the development of communication skills.	High satisfaction reported among students due to the immersive and realistic interaction with VSP, which helps in better preparation for actual patient encounters.
Article title	Summary	Software/AI used	Participants	Control and other groups	Hospital Area	Learning Impact	Student Satisfaction
A Study to Design a Learning Tool "Virtual Patient" for Functional Diagnosis and Clinical Reasoning of Respiratory Dysfunction in the Undergraduate Physiotherapy Curriculum	The study developed and designed a software-based virtual patient for learning functional diagnosis with clinical reasoning of respiratory dysfunction. It assessed and analyzed the need for virtual patient simulation through interviews, designed a respiratory case scenario, and evaluated the utility of the prototype in teaching physiotherapy students.	Oculus Quest	91 students and 15 faculty members	No explicit control group mentioned, but the study involves feedback from both faculty and students regarding the use of the virtual patient in their curriculum.	Physiotherapy at Ravi Nair Physiotherapy College, Datta Meghe Institute of Medical Sciences	The virtual patient scenario helps in developing functional diagnosis and clinical reasoning skills. It is structured and organized, which aids in the quick understanding of diseases.	The feedback analysis from students and faculty suggests that the virtual patient scenario is engaging, interesting, and provides a valuable learning experience.
A Study to Design a Learning Tool "Virtual Patient" for Functional Diagnosis and Clinical Reasoning of Respiratory Dysfunction in the Undergraduate Physiotherapy Curriculum	The study developed a virtual patient in a VR platform to enhance the teaching and learning of respiratory dysfunction diagnosis and clinical reasoning in physiotherapy education. Using Oculus Quest, the virtual environment allowed students to interact with a simulated patient, enhancing learning through structured scenarios that integrated various medical examinations and diagnoses.	Oculus Quest	106 (91 students, 15 faculty members)	No control group was mentioned. The study included physiotherapy students and faculty members only.	Physiotherapy at Datta Meghe Institute of Medical Sciences	- Enhances clinical reasoning skills - Improves clinical judgment and decision-making abilities - Provides organized and logical sequence learning	High; the study reported positive feedback from both students and faculty on the utility of the virtual patient system in learning clinical procedures.
A Study to Design a Learning Tool "Virtual Patient" for Functional Diagnosis and Clinical Reasoning of Respiratory Dysfunction in the Undergraduate Physiotherapy Curriculum	This study aimed to develop a virtual patient software tool to aid in the learning of functional diagnosis and clinical reasoning concerning respiratory dysfunction. It involved the creation of a virtual environment using Oculus Quest, where physiotherapy students could interact with a virtual patient. The study assessed the utility of this tool in teaching by gathering feedback from students and faculty, which indicated that the tool effectively enhanced learning and clinical reasoning skills.	Oculus Quest	98 students and 15 faculty members	Not specified	Physiotherapy	The use of the virtual patient tool improved students' clinical reasoning and decision-making skills. It is structured and organized, enhancing the understanding of diseases.	High, with feedback showing that it was engaging, interesting, and a convenient tool once trained.
Article title	Summary	Software/AI used	Participants	Control and other groups	Hospital Area	Learning Impact	Student Satisfaction
Learning and clinical reasoning experience of second-year medical pharmacology students and teachers with virtual patients developed using OpenLabyrinth	The study explores the perceived usefulness of a virtual case scenario developed and delivered through the OpenLabyrinth platform among medical students and teachers in India. It focused on enhancing clinical reasoning and decision-making skills using virtual patients. This educational tool was well received, especially for its real-life decision-making simulations, though some students expected more on pharmacotherapy.	OpenLabyrinth	19 students and 12 teachers	No control group; the study was a single group observational study.	Medical pharmacology	1. Enhanced readiness for real-life patient care. 2. Improved understanding of interdisciplinary roles. 3. Increased engagement in clinical reasoning.	High; both students and teachers found the virtual scenarios authentic and engaging.
Learning and clinical reasoning experience of second-year medical pharmacology students and teachers with virtual patients developed using OpenLabyrinth	This study explored the effectiveness of virtual patients developed using OpenLabyrinth software to enhance clinical reasoning among second-year medical pharmacology students and teachers. It focused on providing a realistic clinical decision-making environment that mimics real-life medical situations. Participants, both students and teachers, reported that the virtual scenarios effectively mimicked real-life decision-making processes and provided a risk-free environment to practice clinical skills. Feedback indicated that while the tool was well-received and beneficial for understanding patient care from multiple professional perspectives, there were suggestions for improvements in the inclusion of pharmacotherapy aspects and the clarity of user interface navigation.	OpenLabyrinth	19 students and 12 teachers	Not applicable, as this was not a comparative study but a feedback-oriented pilot study on the use of a virtual patient system.	Pharmacology (Medical College)	- Participants felt it provided a realistic simulation of clinical decision-making. - Highlighted the potential for repeated, risk-free practice. - Feedback indicated improved readiness for real-life clinical scenarios among some participants.	- High overall satisfaction with the realism and educational potential of the virtual patients. - Some students expressed a need for better integration of pharmacotherapy and clearer navigational cues in the software.
Learning and clinical reasoning experience of second-year medical pharmacology students and teachers with virtual patients developed using OpenLabyrinth	This study evaluated the use of virtual patient case scenarios on the OpenLabyrinth platform among second-year medical pharmacology students and teachers. Participants interacted with a clinical scenario and provided feedback on its authenticity, engagement, and learning impact. Feedback was positive, highlighting the relevance of the scenarios to real-life medical decision-making.	OpenLabyrinth	31 (19 students and 12 teachers)	No control group was mentioned. All participants engaged with the same virtual case scenario, which was intentionally devoid of pharmacotherapy aspects to avoid bias.	Pharmacology	1. Improved understanding of patient interaction. 2. Exposure to multiple perspectives. 3. Emphasized ethical and behavioral aspects of care. 4. Highlighted the importance of communication in patient management.	74% of students and all teachers found the experience worthwhile. Students noted the lack of pharmacotherapy content, while teachers valued the case for illustrating real-life medical scenarios and emphasizing communication skills.
Article title	Summary	Software/AI used	Participants	Control and other groups	Hospital Area	Learning Impact	Student Satisfaction
Developing an Artificial Intelligence-Based Representation of a Virtual Patient Model for Real-Time Diagnosis of Acute Respiratory Distress Syndrome	The article discusses the development of a deep learning-based surrogate model for real-time diagnosis of Acute Respiratory Distress Syndrome (ARDS) using the Nottingham Physiology Simulator. The process involved using high-performance computing (HPC) to create lightweight models that efficiently simulate ARDS progression, achieving accuracy comparable to more complex systems. The project demonstrated the utility of HPC in rapidly developing and deploying diagnostic support tools in clinical settings.	Nottingham Physiology Simulator; MATLAB, Python, TensorFlow, Keras, Ray Tune	Not specified	Not mentioned	Intensive Care Unit (ICU)	The surrogate model developed mimics the performance of the original NPS, showing high predictive accuracy and enabling faster diagnosis, thus potentially improving treatment outcomes.	Not mentioned
A Study to Design a Learning Tool "Virtual Patient" for Functional Diagnosis and Clinical Reasoning of Respiratory Dysfunction in the Undergraduate Physiotherapy Curriculum	The study aimed to develop a virtual patient for diagnosing respiratory dysfunction and teaching clinical reasoning in undergraduate physiotherapy. It involved creating a virtual case scenario using VR (Oculus Quest) and obtaining feedback from students and faculty. The virtual patient helped improve clinical reasoning, decision-making skills, and diagnosis, based on organized patient interactions in a simulated environment. Faculty and students found it engaging, recommending its inclusion in the curriculum.	Oculus Quest, IBM Watson	98 students, 15 faculty members	No specific control group. The study included faculty and final-year physiotherapy students who tested the virtual patient and provided feedback.	Physiotherapy	Improved clinical reasoning, clinical judgment, decision-making skills, logical sequencing, and better understanding of respiratory dysfunctions.	Yes, students reported high levels of satisfaction, with 80% agreeing it was more engaging than traditional methods.
A Study to Design a Learning Tool "Virtual Patient" for Functional Diagnosis and Clinical Reasoning of Respiratory Dysfunction in the Undergraduate Physiotherapy Curriculum	The study aimed to develop a virtual patient tool for teaching functional diagnosis and clinical reasoning for respiratory dysfunction in physiotherapy students. It was designed using virtual reality (VR) on Oculus Quest. Faculty and student feedback was gathered to evaluate its educational impact. The results indicated that the virtual tool is effective in developing clinical skills and could be a valuable teaching aid.	Oculus Quest and IBM Watson	98 students, 15 faculty members, and 14 subject experts	No specific control group mentioned. Participants included subject experts, students, and faculty members.	Physiotherapy	- Develops clinical reasoning skills. - Enhances clinical judgment. - Facilitates decision-making skills.	Majority of students and faculty expressed positive feedback. Students found it engaging, realistic, and helpful in clinical learning.

GPT4o Responses

Article title	Summary	Software/AI used	Software/AI Description	Participants	Control and other groups	Hospital Area	Learning Impact	Student Satisfaction
Transformative Learning through Virtual Patient Simulations: Predicting Critical Student Reflections	This study explores the impact of virtual patient simulations on transformative learning in nursing education. Using the Digital Clinical Experience (DCE) from Shadow Health, the study analyzes self-reflections of 130 Master of Science in Nursing (MSN) students. The results show that critical self-reflection, indicating transformative learning, is predicted by the number of secondary clinical items discovered by students during their virtual patient interviews.	Digital Clinical Experience (DCE)	A web-based virtual patient simulation software that allows nursing students to interview, examine, document, and reflect on virtual patient interactions.	130 MSN students	No specific control group; study focused on different levels of clinical findings discovered by students.	Nursing	- Encourages deep, critical self-reflection<br>- Promotes clinical reasoning skills<br>- Enhances ability to apply knowledge to different contexts	Not specifically mentioned, but implications for practice suggest positive impacts on student engagement and learning.
Transformative Learning through Virtual Patient Simulations: Predicting Critical Student Reflections	This study examines the impact of virtual patient simulations on transformative learning among nursing students. It utilized the Shadow Health Digital Clinical Experience (DCE) to allow students to interact with virtual patients and perform clinical tasks. The study analyzed self-reflection scores to determine how effectively the simulation facilitated critical reflection and transformative learning. It found that deeper exploration of secondary clinical items led to higher levels of critical reflection.	Shadow Health Digital Clinical Experience (DCE)	A web-based virtual patient simulation software that allows nursing students to interview, examine, and reflect on virtual patients.	130 MSN students	No control group; analyzed performance within a single group of students using the DCE	Nursing	1. Enhanced critical self-reflection 2. Improved clinical reasoning skills 3. Greater engagement in self-assessment	Not explicitly mentioned
Transformative Learning through Virtual Patient Simulations: Predicting Critical Student Reflections	This study investigates the impact of the Shadow Health Digital Clinical Experience (DCE) on nursing students' self-reflection and learning. The research evaluates how the number of clinical items discovered during virtual patient interviews predicts the quality of critical self-reflections. It employs a mixed-methods design to analyze students' reflections and their correlation with simulation performance. Results indicate that discovering secondary clinical items is crucial for deeper self-reflection and transformative learning.	Shadow Health Digital Clinical Experience (DCE)	A web-based virtual simulation software that allows nursing students to interview, examine, and reflect on their experience with virtual patients. It includes various modules for different health assessments and provides structured self-reflection prompts.	130 MSN students	No control group; comparison based on different levels of clinical item discovery	Nursing	- Encourages deeper exploration of patient history.<br>- Promotes critical self-reflection and transformative learning.<br>- Enhances clinical reasoning and application to practice.	Not explicitly mentioned
Article title	Summary	Software/AI used	Software/AI Description	Participants	Control and other groups	Hospital Area	Learning Impact	Student Satisfaction
Hospital nurses' management of agitation in older cognitively impaired patients: do they recognise pain-related agitation?	This study investigates how hospital nurses manage agitation in older cognitively impaired patients experiencing pain. Using virtual simulation, the study aimed to identify the gap in nurses' recognition and treatment of pain-related agitation. The results indicated that a majority of nurses administered antipsychotics instead of treating pain, with only 4.7% correctly identifying and managing the agitation as pain-related.	Virtual Human Toolkit	A suite of modules and tools for creating interactive virtual human conversational agents developed by the Institute of Creative Technologies at the University of Southern California.	274 registered medical and surgical nurses from 10 public hospitals in Queensland, Australia.	No specific control group. The study involved different groups of nurses based on demographics, seniority, workplace, training, experience, and gerontology-specific knowledge.	Medicine	1. Identification of a significant gap in recognizing and managing pain-related agitation.<br>2. Highlighting the need for better training and awareness among nurses to address pain as a potential cause of agitation.	Not explicitly mentioned. The study focused more on the effectiveness of simulation and the gap in clinical practice.
Hospital nurses' management of agitation in older cognitively impaired patients: do they recognise pain-related agitation?	This study investigates how hospital nurses manage agitation in older cognitively impaired patients, particularly if they recognize pain as a cause of agitation. Using a virtual simulation of a patient with dementia, the study observed that most nurses defaulted to using antipsychotics rather than recognizing and treating pain. The simulation included branching pathways, video scenarios, and an avatar to mimic real-life interactions. The results highlighted significant gaps in practice, with only 4.7% of nurses recognizing pain-related agitation.	Virtual Human Toolkit	A suite of modules from the Institute of Creative Technologies at the University of Southern California, designed for creating interactive virtual human conversational agents.	274 registered nurses	No control group mentioned. Groups based on hospital units: medical, surgical, dementia, and delirium units.	Nursing (medical and surgical settings)	- Most nurses missed pain as a cause of agitation. - Predominant reliance on antipsychotics. - Need for improved pain recognition training.	Not explicitly mentioned in the extracted text.
Hospital nurses' management of agitation in older cognitively impaired patients: do they recognise pain-related agitation?	The study investigates how hospital nurses manage agitation in older cognitively impaired patients with pain. It highlights the prevalent use of antipsychotics by nurses, often without recognizing pain as a cause of agitation. The research utilizes virtual simulation to assess nurses' decision-making and identifies gaps in practice, with only 4.7% of nurses recognizing pain-related agitation.	Virtual simulation	A validated vignette-based simulation including branching pathways, video scenarios, and an interactive avatar.	274 registered medical and surgical nurses	No control group mentioned; study includes nurses from different units such as dementia and surgical units	Medical and surgical units	- Revealed a gap in practice regarding pain recognition<br>- Identified reliance on antipsychotics	Not mentioned
Article title	Summary	Software/AI used	Software/AI Description	Participants	Control and other groups	Hospital Area	Learning Impact	Student Satisfaction
User Response to the Simulation of a Virtual Patient with Cranial Nerve Injury	The study evaluates the Neurological Examination Rehearsal Virtual Environment (NERVE) tool, which simulates a virtual patient with cranial nerve injury using speech recognition and a Nintendo Wiimote®. It assesses user reception, ability to identify cranial nerve lesions, and overall satisfaction among medical students, residents, and clinicians.	NERVE, Nintendo Wiimote®	NERVE simulates a life-size virtual patient for cranial nerve exams, utilizing speech recognition and motion tracking with a Nintendo Wiimote® as a virtual hand, ophthalmoscope, and eye-chart.	9 clinicians, 7 residents, 8 MS3 and 4s, 20 MS2s, and 25 MS1s	No control group, various groups of medical students and residents	Medicine	- Identified cranial nerve deficits - Practiced history taking and examination skills - Residents showed the most significant need for repeat exposure	Overall positive reception, residents found it most resembling an actual patient experience despite having the lowest correct identification rates
User Response to the Simulation of a Virtual Patient with Cranial Nerve Injury	The study evaluates the reception, ability to identify CN lesions, and preference of the NERVE tool among medical students, residents, and clinicians. NERVE simulates a virtual patient with cranial nerve deficits using speech recognition and a Nintendo Wiimote®. The study involved participants performing a CN examination, with results showing no significant differences in performance, but indicating educational value. Residents had the lowest correct identification rates, suggesting they might benefit the most from repeated exposure.	NERVE (Neurological Examination Rehearsal Virtual Environment)	A virtual patient system simulating a life-sized patient with cranial nerve deficits. It uses speech recognition and motion tracking with a Nintendo Wiimote® to act as a virtual hand, ophthalmoscope, or eye chart during a simulated medical interview and examination.	69 participants: 9 clinicians, 7 residents, 8 MS3 and 4s, 20 MS2s, 25 MS1s	No control group; groups include clinicians, residents, and medical students across different years	Medicine	1. Improved practical clinical skills<br>2. Consistent evaluation<br>3. Potentially beneficial for repeated exposure, especially for residents	Overall positive but not overwhelming; frustrations with technical limitations; residents had highest approval for overall experience
User Response to the Simulation of a Virtual Patient with Cranial Nerve Injury	This study evaluates the responses of medical students, residents, and clinicians using the Neurological Examination Rehearsal Virtual Environment (NERVE), a cranial nerve exam simulator. It assesses introductory reception, the ability to identify the cranial nerve lesion, and user preference. The study involved participants performing a cranial nerve examination on a virtual patient (VP) using speech recognition and a Nintendo Wiimote®. Results indicated no significant differences in exam performance, technology usage, or satisfaction, though medical students and clinicians were better at identifying the correct deficit compared to residents.	NERVE	A life-size virtual patient (VP) system that uses speech recognition and a Nintendo Wiimote® as a virtual hand, ophthalmoscope, and eye chart.	69 participants: 9 clinicians, 7 residents, 8 MS3 and 4s, 20 MS2s, and 25 MS1s	No control group. Other groups: Clinicians, Residents, Medical Students (MS1, MS2, MS3, and MS4)	Medicine	- Improvement in practical clinical skills (history taking and physical examination). - Increased exposure to cranial nerve deficits. - Enhanced ability to perform cranial nerve exams.	Mentioned. Overall positive experience but varied across groups. Residents had the highest approval of the experience, whereas medical students and clinicians were better at identifying the correct deficit.
Article title	Summary	Software/AI used	Software/AI Description	Participants	Control and other groups	Hospital Area	Learning Impact	Student Satisfaction
Developing a Conversational Virtual Standardized Patient to Enable Students to Practice History-Taking Skills	This study describes the development and evaluation of a virtual standardized patient (VSP) system where medical students practice history-taking and diagnostic skills using natural language interactions. The VSPs are created using ChatScript for dialogue management and Unity for 3D character simulation. The study found that VSPs effectively helped students practice and improve their history-taking skills, with high accuracy in student-VSP interactions. Future improvements include automatic assessment modules for immediate feedback.	ChatScript, Unity	ChatScript: an NLP engine for managing doctor-patient dialogues. Unity: a high-fidelity game engine for creating 3D characters.	Third-year students: 18, First-year students: 34	Different levels of medical students, no specific control group mentioned.	Medicine, General Internal Medicine	- Improved accuracy in history-taking skills.<br>- Ability to develop appropriate differential diagnoses.<br>- Enhanced practice before real patient interactions.	Not specifically mentioned.
Developing a Conversational Virtual Standardized Patient to Enable Students to Practice History-Taking Skills	This study discusses the development and implementation of a Virtual Standardized Patient (VSP) system that uses natural language processing to allow medical students to practice history-taking skills. The VSP system integrates ChatScript for dialogue management and Unity 3D for creating responsive 3D characters. The system was tested with medical students at Ohio State University College of Medicine, showing high accuracy in response and differential diagnosis.	ChatScript, Unity 3D	ChatScript: Open-source natural language processing engine for managing doctor-patient dialogues. Unity 3D: High-fidelity game engine for creating 3D characters.	52 students (18 third-year and 34 first-year students)	No control group mentioned. Groups: Third-year and first-year students.	General Internal Medicine, Family Medicine, Emergency Medicine	- High accuracy in developing differential diagnoses (79%-86%) - Effective practice for history-taking before real patient encounters - Enhanced learning of clinical decision-making skills	Not explicitly mentioned.
Developing a Conversational Virtual Standardized Patient to Enable Students to Practice History-Taking Skills	The study developed a Virtual Standardized Patient (VSP) system allowing medical students to practice history-taking skills through natural language interactions. This system integrates ChatScript for dialogue management and Unity for creating emotionally responsive 3D characters. The study, conducted at Ohio State University College of Medicine, demonstrated that students could take histories, develop differential diagnoses, and document encounters accurately.	ChatScript, Unity	ChatScript is an open-source NLP engine used for managing doctor-patient conversations. Unity is a high-fidelity game engine used to create emotionally responsive 3D characters.	52 students (18 third-year and 34 first-year)	No explicit control group. First-year and third-year students were tested separately with different case complexities.	General Internal Medicine	1. Improved history-taking skills.<br>2. Accurate development of differential diagnoses.<br>3. Enhanced documentation skills in an EMR.	Not explicitly mentioned
Article title	Summary	Software/AI used	Software/AI Description	Participants	Control and other groups	Hospital Area	Learning Impact	Student Satisfaction
Affect and Cognitive Closure in Students—A Step to Personalised Education of Clinical Assessment in Psychology with the Use of Simulated and Virtual Patients	The study investigates the impact of affect and cognitive closure on learning performance using simulated patients (SP) and virtual patients (VP) among 56 fifth-year clinical psychology students. The study aims to measure the effectiveness of SP and VP in teaching psychological interview techniques and clinical reasoning. The results indicate that SP was more effective for interview skills, while VP was beneficial for most clinical reasoning skills. The findings suggest that affect and cognitive closure can be used to tailor personalized education for psychology students.	IBM SPSS 25	Statistical analysis software used for data analysis	56 fifth-year clinical psychology students	No control group; participants were divided into SP and VP groups	Clinical psychology	- SP improved interview skills. - VP enhanced clinical reasoning skills. - SP beneficial for high EFNC students. - VP beneficial for low EFNC students.	Yes, detailed analysis showed positive feedback on both SP and VP methods, with specific preferences based on EFNC levels.
Affect and Cognitive Closure in Students—A Step to Personalised Education of Clinical Assessment in Psychology with the Use of Simulated and Virtual Patients	This study investigated the learning performance of fifth-year clinical psychology students using simulated patients (SP) and virtual patients (VP) to enhance clinical assessment skills. It examined the roles of affect and cognitive closure in developing interview and clinical reasoning skills. SP was found to be more effective for interview skills, while VP was beneficial for clinical reasoning. The results suggest that personalizing education based on these psychological variables can enhance student performance.	VP computer program	A computer-based tool that assesses clinical interview skills through short recordings of diagnostic psychological consultations, providing feedback and assessing students' clinical reasoning skills.	56 fifth-year clinical psychology students	SP group (25 initial, 24 final), VP group (31 initial, 27 final)	Psychology	SP improved interview skills; VP enhanced most clinical reasoning skills; VP provided structure for students with low EFNC, while SP helped those with high EFNC build on structuring skills.	Not explicitly mentioned
Affect and Cognitive Closure in Students—A Step to Personalised Education of Clinical Assessment in Psychology with the Use of Simulated and Virtual Patients	The study analyzes the impact of affect and cognitive closure on the learning performance of clinical psychology students using simulated patients (SP) and virtual patients (VP). It was found that SPs were more effective for interview skills, while VPs were more beneficial for clinical reasoning skills. The study highlights the importance of personalized education based on psychological variables.	VP and SP tools	The VP tool is a computer program that simulates patient interactions, while the SP tool involves role-playing by trained individuals. Both tools are used to teach and measure psychological interview techniques and clinical reasoning.	56 fifth-year clinical psychology students	SP group and VP group	Psychology	- SPs improved interview skills. - VPs enhanced clinical reasoning skills. - VP training helped students with low EFNC by providing structure. - SP training benefited students with high EFNC by allowing them to utilize advanced structuring skills.	Not mentioned
Article title	Summary	Software/AI used	Software/AI Description	Participants	Control and other groups	Hospital Area	Learning Impact	Student Satisfaction
Using a Virtual Patient via an Artificial Intelligence Chatbot to Develop Dental Students' Diagnostic Skills	The study evaluates the use of an AI chatbot, named Julia, as a virtual patient to help dental students at the Universidad Europea de Madrid improve their diagnostic skills for pulp pathology. The study involved fourth and fifth-year dental students who interacted with Julia via Telegram over several weeks and then completed a survey. Results showed high satisfaction among students, particularly those in their fifth year, and those who made a correct diagnosis rated the technology more positively.	Dialogflow	Dialogflow® is an intuitive AI application used to create conversational chatbot flows capable of understanding human language nuances through natural language processing and machine learning.	193 students (58 fourth-year, 135 fifth-year)	No explicit control group mentioned. Groups compared by year (fourth vs. fifth-year) and sex.	Dentistry	- Improved communication skills - Increased confidence and security - Enhanced professionalism - Effective in completing diagnostic questions - High recommendation for inclusion in the curriculum	Yes, high satisfaction reported, particularly by fifth-year students, with a mean satisfaction score of 4.36.

Using a Virtual Patient via an Artificial Intelligence Chatbot to Develop Dental Students' Diagnostic Skills	The study explores the implementation of an AI chatbot, named Julia, as a virtual patient to enhance the diagnostic skills of fourth and fifth-year dental students. The chatbot simulates a patient with reversible pulpitis, engaging students in diagnostic dialogues. The study measures the satisfaction and perceived effectiveness of the chatbot through a survey. Results indicate high satisfaction among students, particularly fifth-year students, who found the tool more beneficial.	Dialogflow	Dialogflow is an intuitive AI application capable of understanding and processing natural language to create conversational flows for chatbots.	193 dental students (109 females, 84 males)	No control group; fourth and fifth-year student groups	Dentistry	- Enhances communication skills - Increases confidence and security in patient interactions - Promotes professional growth	High satisfaction reported, with fifth-year students showing higher satisfaction (mean score 4.36) compared to fourth-year students.
Using a Virtual Patient via an Artificial Intelligence Chatbot to Develop Dental Students' Diagnostic Skills	This study assesses student satisfaction after interacting with an AI-based virtual patient chatbot named Julia. The study involved 193 fourth and fifth-year dental students at the Universidad Europea de Madrid. The results showed high satisfaction levels, particularly among fifth-year students. The study suggests incorporating AI chatbots in dental curricula to improve diagnostic skills and adapt to new technological advancements.	Artificial Intelligence (AI) Chatbot	A conversational chatbot named Julia, created using AI to simulate a virtual patient suffering from reversible pulpitis, enabling interactive diagnostic practice for dental students.	193 students	No control group specified	Dentistry	1. Improved diagnostic skills 2. Positive evaluation of AI integration 3. Enhanced confidence in clinical practice	High satisfaction reported, with a mean score of 4.36. Fifth-year students expressed higher satisfaction than fourth-year students.
Article title	Summary	Software/AI used	Software/AI Description	Participants	Control and other groups	Hospital Area	Learning Impact	Student Satisfaction
Learning clinical reasoning with virtual patients	The article addresses the challenge of teaching clinical reasoning by introducing a virtual patient (VP) system that uses artificial intelligence (AI) and natural language processing. This system allows medical students to interact with a simulated patient in real-time, enhancing their clinical reasoning skills through practice and feedback. The study was conducted with 112 medical students at Kaohsiung Medical University and E-Da Hospital in Taiwan. The findings indicated high appreciation among students for the VP system, with reported increases in confidence, proficiency, and organized thought processes.	AI and Natural Language Processing	A virtual patient with AI and natural language processing capabilities that recognizes and responds to students' verbal questions, providing real-time feedback and modifying responses based on students' decisions.	112 medical students	Not specified	Medicine	- Early clinical exposure - Repetitive practice with feedback - Improved knowledge structure - Increased confidence and proficiency	Yes, up to 80% of students reported increased confidence and proficiency, and found the learning interesting
Learning clinical reasoning with virtual patients	This study developed a virtual patient (VP) system utilizing artificial intelligence (AI) and natural language processing (NLP) to enhance clinical reasoning skills in medical students. The VP simulates clinical encounters where students can ask questions, request exams, and receive real-time feedback. The system provides instant performance scores and feedback based on expert-defined standards.	Artificial intelligence and natural language processing	A VP system that recognizes and responds to student queries in real-time, providing multimedia feedback and performance scores.	112 medical students	No control group mentioned. Only the experimental group using the VP system.	Pediatrics	1. Early clinical exposure 2. Repetitive practice with immediate feedback 3. Self-directed learning 4. Improved knowledge structure	High satisfaction reported with 80% of students noting increased confidence and proficiency. Students found the learning experience interesting and wanted more VP-based simulations.
Learning clinical reasoning with virtual patients	The article discusses the development and implementation of a virtual patient (VP) system utilizing AI and natural language processing to enhance clinical reasoning skills in medical students. The system simulates real-life patient encounters, allowing students to practice making diagnoses and receive instant feedback. Field testing involved 112 students from Kaohsiung Medical University and E-Da Hospital. The VP system provides early clinical exposure, promotes self-directed learning, and improves students' competence and confidence in clinical problem-solving.	Virtual patient (VP) with artificial intelligence and natural language processing	The VP system recognizes and responds to students' verbal questions in real-time, providing multimedia responses for physical examinations, lab tests, and imaging studies. It displays expert-derived schemata and generates performance scores with feedback.	112 medical students	No control group mentioned. Field testing only involved medical students.	Medicine	1. Early clinical exposure to patients<br>2. Repetitive practice with feedback<br>3. Improved knowledge structure<br>4. Increased confidence and proficiency in real-life patient scenarios	Yes, high appreciation was reported with 80% of users noting increased confidence and proficiency. Students found the learning experience interesting and expressed a desire for more VP-based training.
Article title	Summary	Software/AI used	Software/AI Description	Participants	Control and other groups	Hospital Area	Learning Impact	Student Satisfaction
The virtual patient as a learning tool: a mixed quantitative qualitative study	This study evaluates the effectiveness of a low-fidelity virtual patient (VP) simulator in developing clinical reasoning and history-taking skills among undergraduate medical students. A course using the VP as the primary teaching tool was conducted, and student performance was assessed through a mix of quantitative and qualitative methods before and after the course. Significant improvements were observed in all evaluated aspects.	Virtual Patient (VP)	A web-based tool that simulates real clinical scenarios, allowing students to interact through typed questions and access physical exam findings.	20 undergraduate medical students	No control group mentioned. The study involved the same group of students evaluated before and after the intervention.	Medicine	- Significant improvement in interview skills, physical exams, clinical judgment, relevance of medical exams, and case presentation. - Enhanced logical and structured thinking processes. - Increased ability to review and reassess information.	Yes. Students found the VP to be an easy-to-use, motivating, and stress-free learning tool, especially useful at the beginning of their careers. They appreciated the ability to make mistakes without real consequences.
The virtual patient as a learning tool: a mixed quantitative qualitative study	This study investigates the effectiveness of a low-fidelity simulator called Virtual Patient (VP) in developing clinical reasoning and history-taking skills in undergraduate medical students. The study employed a quasi-experimental design with pre- and post-course assessments involving standardized patients. The VP tool allowed students to engage in interactive clinical scenarios and was found to improve their skills significantly.	Virtual Patient (VP)	A web-based tool that simulates real clinical scenarios for educational purposes. It allows professors to design clinical cases and students to interact through questions, physical exam findings, and additional studies.	20 undergraduate medical students	No control group specified; all participants used the VP tool within the course.	Medicine	Significant improvement in history taking, clinical reasoning, physical exams, relevance of medical exams, and case presentation.	High; students found the VP tool easy to use, motivating, and helpful in creating logical and structured processes without the stress of real patient interaction.
The virtual patient as a learning tool: a mixed quantitative qualitative study	This study evaluated the effectiveness of a low-fidelity simulator, the Virtual Patient (VP), in developing clinical reasoning and history-taking skills among undergraduate medical students. Using a quasi-experimental design, the study measured students' skills before and after the course, revealing a significant improvement. The VP was perceived as a motivating, stress-free, and easy-to-use tool that allowed students to learn without fear of making mistakes.	Virtual Patient (VP)	A Web-based tool that simulates real clinical scenarios, allowing professors to design clinical cases for teaching purposes. It helps students interact with virtual patients through typed questions, physical exam findings, and additional studies.	20	No control group; single group of undergraduate medical students.	Medicine	- Significant improvement in interview, physical exam, clinical judgment, relevance of medical exams, and case presentation skills.<br>- Encouraged logical and structured processes.<br>- Allowed practice without consequences and enabled review and reassessment of information.	Yes, students found the VP motivating and easy to use, especially early in their careers.
Article title	Summary	Software/AI used	Software/AI Description	Participants	Control and other groups	Hospital Area	Learning Impact	Student Satisfaction
Evaluation of an interactive virtual patient system (ISP) in an OSCE clinical assessment	The study evaluates the use of an Interactive Simulation of Patients (ISP) in an OSCE exam with 118 fourth-year medical students. The ISP system was integrated as one of the twelve examination modules to assess clinical reasoning and problem-solving abilities. Results showed that the ISP could differentiate between students' performances, though the presence of assistants affected outcomes.	Interactive Simulation of Patients (ISP)	A comprehensive virtual patient-based learning tool designed to help medical students practice clinical reasoning skills, resembling a realistic patient encounter.	118 students	Two subgroups per hospital; groups compared within each hospital	Medicine	- Differentiated student performance - Enhanced clinical reasoning skills	- 63% found the system engaging - 78% found it realistic
The use of a virtual patient case in an OSCE-based exam – A pilot study	This pilot study aimed to evaluate the potential of using a virtual patient (VP) case in an Objective Structured Clinical Examination (OSCE) for assessing clinical reasoning and problem-solving skills among medical students. The study involved 110 fourth-year medical students from four teaching hospitals. The results indicated that VP systems could differentiate student performance effectively and had a positive impact on student engagement and realism in clinical scenarios. However, the presence of human assistants influenced outcomes, highlighting the need for standardized training and scoring rubrics.	Interactive Simulation of Patients (ISP)	ISP is a comprehensive virtual patient-based learning tool designed to help medical students practice clinical reasoning skills. It allows students to take medical history, perform physical examinations, request lab tests, and suggest diagnoses in an unscripted, authentic case interaction.	110	No control group mentioned.	Medicine	1. Enhanced ability to solve clinical cases. 2. Improved clinical reasoning skills. 3. Better engagement with realistic scenarios.	Yes, 78% found the VP engaging and 63% found it realistic. Most students saw potential in using VP systems in future exams.
The use of a virtual patient case in an OSCE-based exam – A pilot study	This study evaluates the use of a virtual patient (VP) system called ISP in an OSCE-based exam. It aimed to assess the students' clinical reasoning and problem-solving skills. Results indicate that VP systems can reliably differentiate student performances but also highlight the influence of human assistants on outcomes. Training and validation of assessment tools are necessary for future use.	Interactive Simulation of Patients (ISP)	ISP is a high-fidelity virtual patient system designed for medical students to practice clinical reasoning through realistic patient interactions, including history taking, physical exams, and lab tests.	110 students (118 enrolled, 8 were excused)	Yes, students were divided into subgroups at four hospitals (H1, H2, H3, H4) and rotated through different stations including ISP and standardized patients.	Medicine (surgery course)	1. Differentiates student performance effectively. 2. Positive impact of high-quality assistance. 3. Engaging and realistic learning tool.	Mixed; overall opinions of the surgery course and OSCE exam rated higher than the ISP program. Students from some hospitals rated ISP lower due to first-time use and quality of assistance.
Article title	Summary	Software/AI used	Software/AI Description	Participants	Control and other groups	Hospital Area	Learning Impact	Student Satisfaction
A Study to Design a Learning Tool "Virtual Patient" for Functional Diagnosis and Clinical Reasoning of Respiratory Dysfunction in the Undergraduate Physiotherapy Curriculum	The study aimed to develop a software-based "virtual patient" for teaching functional diagnosis and clinical reasoning of respiratory dysfunction in undergraduate physiotherapy students. The study assessed the need for such a tool, designed a prototype, and evaluated its effectiveness through feedback from students and faculty. The results indicated that the virtual patient case scenario was well-organized and could effectively be used as a teaching and learning tool.	Oculus Quest	A VR platform used to create an immersive virtual patient environment where students can practice clinical skills using hand-tracking technology.	98 students and 15 faculty members	No specific control group; other groups included faculty members for feedback	Physiotherapy	1. Development of clinical reasoning skills 2. Improved diagnostic reasoning 3. Enhanced problem-solving skills 4. Facilitated analytical thinking	High satisfaction with the virtual patient tool for clinical postings, clinical reasoning skills, and communication skills development
A Study to Design a Learning Tool "Virtual Patient" for Functional Diagnosis and Clinical Reasoning of Respiratory Dysfunction in the Undergraduate Physiotherapy Curriculum	The study aimed to develop a software-based virtual patient for teaching functional diagnosis and clinical reasoning for respiratory dysfunction in physiotherapy students. The study included creating a virtual environment using Oculus Quest and assessing its utility based on feedback from students and faculty. Results suggested that the virtual patient scenario was organized, effective as a teaching modality, and well-received by participants.	Blender, IBM Watson Model, Unity	Blender was used for modeling and animation, IBM Watson Model for text-to-speech interaction, and Unity for assembling the virtual environment	98 students and 15 faculty members	Single group with feedback collection from both students and faculty	Physiotherapy	Improved clinical reasoning, diagnosis skills, analytical thinking, communication skills	High satisfaction; 56% found it easy to use, 64% felt it enhanced understanding, 57% supported its inclusion in the curriculum
A Study to Design a Learning Tool 'Virtual Patient' for Functional Diagnosis and Clinical Reasoning of Respiratory Dysfunction in the Undergraduate Physiotherapy Curriculum	This study aimed to develop and design a software-based "virtual patient" for learning functional diagnosis and clinical reasoning of respiratory dysfunction. The study involved need analysis, design of a prototype using Oculus Quest, and feedback from faculty and students. The prototype was found to be organized and effective as a teaching and learning modality, suggesting that it could help develop functional diagnosis and clinical reasoning skills in undergraduate physiotherapy students.	Oculus Quest	A VR platform used for creating a virtual environment for the virtual patient case scenario.	98 students and 15 faculty members	No control group specified	Physiotherapy	Improved clinical reasoning skills, clinical judgment, and decision-making skills.	Positive feedback: engaging, interesting, convenient, and helpful in reducing anxiety and fear of dealing with real patients.
Article title	Summary	Software/AI used	Software/AI Description	Participants	Control and other groups	Hospital Area	Learning Impact	Student Satisfaction
Learning and clinical reasoning experience of second-year medical pharmacology students and teachers with virtual patients developed using OpenLabyrinth	The study evaluated the use of virtual case scenarios developed using OpenLabyrinth among 20 second-year medical students and 12 pharmacology teachers. Feedback indicated high perceived authenticity and relevance to real-life medical decision-making. The software allowed for branched decision-making paths in clinical case scenarios, enhancing the learning experience. The study concluded that virtual patient scenarios are well-received and useful in medical education, particularly in resource-limited settings.	OpenLabyrinth	OpenLabyrinth is a server-based software platform that enables the creation and use of interactive virtual patient scenarios. It allows for remote access and includes tools for incorporating multimedia elements and branched decision-making paths.	32 (20 students, 12 teachers)	No control group; only participating students and teachers	Pharmacology	1. Improved understanding of patient interaction<br>2. Enhanced decision-making skills<br>3. Better preparedness for real-life scenarios	Yes, positive feedback from both students and teachers
Learning and clinical reasoning experience of second-year medical pharmacology students and teachers with virtual patients developed using OpenLabyrinth	The study explores the use of virtual case scenarios using OpenLabyrinth to enhance learning and clinical reasoning among second-year medical pharmacology students and their teachers. It highlights the perceived usefulness and educational impact of these scenarios. The study involved 20 students and 12 teachers, and feedback was gathered on various aspects such as the authenticity of patient encounters and the learning effect. Overall, the study found that virtual case scenarios were positively received and beneficial in providing clinical experience in a resource-limited setting.	OpenLabyrinth	OpenLabyrinth is a server-based software that allows the creation and playing of virtual patient scenarios. It supports multimedia integration and branched decision-making paths, providing a risk-free environment for medical training.	20 second-year medical students and 12 pharmacology teachers	No control group specified.	Pharmacology	- Improved understanding of patient interaction<br>- Enhanced interprofessional collaboration<br>- Provided a broader perspective on patient care<br>- Encouraged proper communication and ethical behavior	Yes, positive feedback from both students and teachers. 95% of students and all faculty felt the virtual scenarios were realistic and educational.

Learning and clinical reasoning experience of second-year medical pharmacology students and teachers with virtual patients developed using OpenLabyrinth	The study explores the perceived usefulness of virtual case scenarios developed and delivered through the OpenLabyrinth platform among second-year medical students and pharmacology teachers. 20 students and 12 teachers participated, accessing the scenario via their devices. Feedback was obtained through a validated questionnaire. 95% of students and all faculty felt the scenarios simulated real-life decision-making. The study concluded that virtual case scenarios are well-received and beneficial for medical education.	OpenLabyrinth	OpenLabyrinth is a server-based application for creating and playing virtual patient scenarios, allowing for branched decision-making paths and multimedia integration.	19 students, 12 faculty members	No control group mentioned	Pharmacology	- Simulated real-life decision-making<br>- Enhanced understanding of patient interaction<br>- Provided a broad clinical perspective	Positive feedback, with high satisfaction from both students and teachers

Supplementary File S2. Summarizing Prompt and GPT Responses

Summarizing Prompt

I am going to send you three summary tables that talk about the same article, unify them into a single table that includes everyone's information, without repeating information or concepts.
Maintain the same structure of the table that I send you.

I will also attach the article so you can make sure everything included is correct and there is no missing information.

GPT4 Responses

Article title	Summary	Software/AI used	Software/AI Description	Participants	Control and other groups	Hospital Area	Learning Impact	Student Satisfaction
Transformative Learning through Virtual Patient Simulations: Predicting Critical Student Reflections	The study analyzes the effect of the amount of dialogue and clinical items discovered in virtual patient simulations on nursing students' performance. It suggests that deeper exploration of secondary clinical items correlates with higher levels of student self-reflection.	Digital Clinical Experience™ (Shadow Health)	Web based simulation software allowing nursing students to interview and examine virtual patients and reflect on their performance.	130 MSN students	The performance of students who explored secondary clinical items was compared with those who found only primary clinical findings.	Nursing Education	Discovering secondary clinical items predicted critical self-reflection, indicating transformative learning. Primary clinical findings alone did not lead to this level of reflection.	Not specified in the article.
Hospital nurses' management of agitation in older cognitively impaired patients: do they recognise pain-related agitation?	This study investigates how hospital nurses manage agitation in older cognitively impaired patients, focusing on whether they recognize agitation caused by pain. Nurses often rely on antipsychotics rather than recognizing and treating the underlying pain. A virtual simulation was used to assess nurses' responses in managing patient agitation without prior information about the patient's pain condition. The findings revealed that most nurses did not identify pain as a cause of agitation and predominantly used antipsychotics for management. The study highlighted a significant gap in recognizing pain-related agitation, suggesting a need for improved training and awareness.	Virtual Human Toolkit	A software suite from the Institute of Creative Technologies that includes tools for creating interactive, conversational virtual human agents. Used to construct a simulation for nurse training, it provides modules for creating interactive virtual human conversational agents. It was used to construct an avatar for the virtual simulation, enabling conversation-like interactions with the participants.	274 (registered) nurses	No specific control group; the study was cross-sectional with multivariate descriptive design and involved various nurse participants undergoing the simulation independently.	Medical and surgical settings in hospitals	The simulation exposed a significant gap in recognizing and managing pain-related agitation, demonstrating an over-reliance on antipsychotics, with only 5% of nurses identifying pain as a source of agitation correctly.	Not explicitly mentioned; however, the simulation's reliability and immersion were rated highly, which could indirectly indicate participant engagement and satisfaction.
User Response to the Simulation of a Virtual Patient with Cranial Nerve Injury	The study evaluated the effectiveness of the Neurological Examination Rehearsal Virtual Environment (NERVE) in training medical students, residents, and clinicians to identify cranial nerve lesions. Despite some technical issues, such as problems with speech recognition, the tool was beneficial for learning, improving identification of cranial nerve lesions, especially with repeated exposure.	NERVE	NERVE is a virtual patient simulation tool that integrates speech recognition and motion tracking via a Nintendo Wiimote®, used as a virtual hand, ophthalmoscope, and eye-chart. Developed by the University of Florida, University of Georgia, and the Medical College of Georgia, it aims to enhance clinical skills training and provide realistic clinical scenarios for self-	69 participants (9 clinicians, 7 residents, 53 medical students)	No specific control group; study appears to involve only one group using the NERVE system.	Used broadly for medical education, specifically in neurology.	Enhanced examination skills, familiarity with neurological exams, and the ability to practice rare clinical scenarios. The tool was particularly useful for residents.	Mixed: Moderate to high overall satisfaction reported. Participants acknowledged the educational benefits despite some technical limitations and varying levels of realism.
Developing a Conversational VSP	This article discusses the development and implementation of a Virtual Standardized Patient (VSP) system designed to train medical students in history-taking skills. The VSP system uses natural language processing to engage students in realistic patient-doctor conversations. The study explores the system's conversational accuracy and its effects on student learning, diagnostic skills, and clinical reasoning.	ChatScript and Unity 3D	ChatScript is an open-source NLP engine for creating conversational AI, suited for narrow but detailed conversations such as medical history-taking. Unity 3D creates immersive 3D environments for these interactions.	52 medical students (18 third-year, 34 first-year) from Ohio State University College of Medicine	Control group of third-year medical students and first-year medical students in different complexity scenarios	Not specified, likely medical education or clinical simulation area	The VSP demonstrated high conversational accuracy (79%-86%) and allowed students to effectively practice and improve their differential diagnosis and clinical reasoning skills.	High satisfaction reported among students, with improvements in preparation for real patient encounters due to the immersive and realistic interaction with the VSP.
Affect and Cognitive Closure in Students—A Step to Personalised Education of Clinical Assessment in Psychology with the Use of Simulated and Virtual Patients	The study examined how affect and cognitive closure impact learning outcomes in clinical psychology using simulated (SP) and virtual patients (VP) among 56 fifth-year students. SPs were found to enhance interview skills effectively, while VPs were beneficial for clinical reasoning skills, particularly for students with low efficacy in fulfilling their need for closure (low EFNC). The research suggests that personalized education approaches can be tailored to individual learning abilities, improving educational outcomes in clinical psychology.	Specific software tools developed by the authors for simulated and virtual patient interactions, aimed at teaching and evaluating psychological interview techniques and clinical reasoning.	The VP software used in the study functions as a structured teaching tool, providing a controlled environment that helps students with low EFNC develop clinical reasoning skills by guiding them through diagnostic processes.	56 fifth-year clinical psychology students participated.	Participants were divided into groups using simulated patients (SP) and virtual patients (VP). There were no explicit control groups mentioned, but the study involved both SP and VP groups, suggesting a comparative approach.	The study was conducted within the academic setting of the psychology department, not in a hospital area.	Simulated patients were more effective for teaching interview skills, while virtual patients were beneficial for clinical reasoning skills, especially for students with lower efficacy in fulfilling their need for closure.	Student satisfaction was not explicitly measured or mentioned in the results. The focus was on learning outcomes rather than satisfaction.
Using a Virtual Patient via an Artificial Intelligence Chatbot to Develop Dental Students' Diagnostic Skills	The study conducted at the Universidad Europea de Madrid evaluated the impact of an AI chatbot, named Julia, on the diagnostic skills of dental students. Julia simulated a patient with reversible pulpitis, providing a controlled environment for students to practice diagnostic skills. The chatbot was accessed via Telegram and integrated into the dental curriculum, aiming to enhance learning through interaction in a stress-free environment. It emphasized repetitive practice and feedback, suggesting the potential for wider curriculum integration and highlighting the importance of adapting to technological advancements in dental education.	Dialogflow®	Dialogflow, a software developed by Google, is used to create conversational user interfaces. It utilizes natural language understanding to comprehend user intent behind text inputs, making it suitable for building interactive chatbots like Julia, designed to simulate dental patient interactions and assist in medical training.	193 fourth and fifth-year dentistry students from the Universidad Europea de Madrid	No specific control group; study participants used the chatbot in a descriptive cross-sectional study format	Dental Education (Dentistry)	Enhanced diagnostic skills and competencies, increased exposure to clinical cases, self-evaluation, and accountability, improved adaptation to technological advancements, and the ability to practice in a risk-free environment.	High satisfaction reported among students with mean ratings around 4.36 on a 5-point scale. The study noted higher satisfaction among 5th-year students, indicating a positive reception and perceived usefulness of the AI tool in their learning process.
Learning clinical reasoning with virtual patients	This study assesses a virtual patient (VP) system integrated with artificial intelligence and natural language processing to enhance medical education in clinical reasoning. The VP system simulates clinical scenarios, allowing students to interact and make decisions, with immediate feedback provided to improve learning outcomes. Tested with 112 medical students at Kaohsiung Medical University and E-Da Hospital in Taiwan, the study aimed to improve clinical competence and confidence in diagnosis.	Virtual patient (VP) system with artificial intelligence and natural language processing	The VP system is designed to simulate real-time interactions between students and patients, improving clinical reasoning skills through immediate feedback and dynamic problem-solving scenarios.	112 medical students	No control group specified	Medicine (Kaohsiung Medical University and E-Da Hospital)	- Facilitates early clinical exposure and self-directed learning.<br>- Offers opportunities for repetitive practice and immediate feedback.<br>- Enhances competence in clinical problem-solving, knowledge structure, and decision-making confidence.	- 80% of students reported increased confidence and proficiency in handling real patients.<br>- Students showed a high appreciation for the system and a desire for more VP-based experiences.
The virtual patient as a learning tool: a mixed quantitative qualitative study	This study evaluated the effectiveness of the Virtual Patient (VP) simulator as a low-fidelity tool for teaching clinical reasoning and history-taking skills to undergraduate medical students at Universidad del Rosario. Using a mixed methods approach, the course integrated VPs in a constructivist learning environment, allowing for practice and refinement of essential medical skills without patient risk. Results indicated significant improvements in students' skills from pre- to post-course assessments and facilitated deeper understanding of medical practices through repetitive, focused practice.	Virtual Patient (Web-based tool)	VP is a web-based tool that allows the creation and modification of clinical scenarios for educational purposes. It enables students to engage in simulated clinical interactions and decision-making without risk to real patients.	20 undergraduate medical students	No specific control group; pre-and-post course evaluations were used for all participants.	Medicine	Significant improvements in:<br>1. Interview skills<br>2. Physical exam skills<br>3. Clinical judgment<br>4. Relevance of medical exams<br>5. Case presentation abilities	High satisfaction among students. Found VP easy to use, motivating, stress-reducing, and beneficial for learning without stress, especially at the beginning of their careers. Helpful in making mistakes without real-world consequences.
The use of a virtual patient case in an OSCE-based exam – A pilot study	This pilot study assessed the integration of a virtual patient (VP) into an OSCE-based exam to measure medical students' clinical reasoning and problem-solving skills at Karolinska Institutet. The VP was used to test clinical skills in a controlled environment, aiming to enhance learning through realistic simulation. The study observed interactions with VPs, the ability to differentiate student performance, and the impact of human assistants on outcomes. It emphasized the need for system familiarity and suggested potential for VPs in high-stakes assessments, with recommendations for further development and student training.	Interactive Simulation of Patients (ISP)	ISP is a high-fidelity virtual patient-based learning tool designed for medical students to practice clinical reasoning skills. It provides an authentic experience by allowing students to navigate freely, take patient histories, perform physical examinations, and suggest diagnoses using extensive interactive functions.	118 fourth-year medical students	No specific control group, but subgroups were made for comparison based on assistance received.	Medicine, within the surgical course context at Karolinska hospitals.	- VP can differentiate student performance reliably.<br>- Provides a realistic and engaging testing scenario.<br>- Emphasizes the need for proper training and system familiarity.<br>- Suggests VPs might be useful for high-stakes exams with further development.	Mixed results: Generally positive, with students finding the VP engaging and realistic. Some students noted concerns with the system's interface and limited interactive dialogue.
A Study to Design a Learning Tool "Virtual Patient" for Functional Diagnosis and Clinical Reasoning of Respiratory Dysfunction in the Undergraduate Physiotherapy Curriculum	The study aimed to design and assess a virtual patient simulation for learning functional diagnosis with clinical reasoning of respiratory dysfunction, involving the development of a virtual patient case scenario on Oculus Quest. The results suggested that the prototype was organized, sequenced, and could replace the need for extensive infrastructure in teaching functional diagnosis and clinical reasoning skills.	Oculus Quest	Utilizing a VR platform, a prototype was designed for immersive interaction with a virtual patient scenario common in clinical postings, allowing for history taking, examination, and diagnosis within a hand-tracking environment.	91 students and 15 faculty members from Ravi Nair Physiotherapy College, Datta Meghe Institute of Medical Sciences	Not specified	Physiotherapy	Enhanced understanding of diseases, facilitated respiratory assessment, diagnosis, and functioning classification training, potentially useful for preclinical exposure.	High satisfaction reported, indicating the VR environment as engaging, interesting, and conducive to learning.
Learning and clinical reasoning experience of second-year medical pharmacology students and teachers with virtual patients developed using OpenLabyrinth	This study explored the effectiveness of virtual patients developed using OpenLabyrinth software to enhance clinical reasoning among second-year medical pharmacology students and teachers. It focused on providing a realistic clinical decision-making environment that mimics real-life medical situations. Participants, both students and teachers, reported that the virtual scenarios effectively mimicked real-life decision-making processes and provided a risk-free environment to practice clinical skills. Feedback indicated that while the tool was well-received and beneficial for understanding patient care from multiple professional perspectives, there were suggestions for improvements in the inclusion of pharmacotherapy aspects and the clarity of user interface navigation.	OpenLabyrinth	OpenLabyrinth is an open-source virtual patient authoring and playing system that allows for the creation and execution of case-based scenarios. It provides a platform for medical training that can simulate various patient interactions and clinical situations, enabling users to make decisions that affect the outcomes of virtual patients.	19 students and 12 teachers	Not applicable, as this was not a comparative study but a feedback-oriented pilot study on the use of a virtual patient system.	Pharmacology (Medical College)	- Participants felt it provided a realistic simulation of clinical decision-making. - Highlighted the potential for repeated, risk-free practice. - Feedback indicated improved readiness for real-life clinical scenarios among some participants.	- High overall satisfaction with the realism and educational potential of the virtual patients. - Some students expressed a need for better integration of pharmacotherapy and clearer navigational cues in the software.
Developing an Artificial Intelligence-Based Representation of a Virtual Patient Model for Real-Time Diagnosis of Acute Respiratory Distress Syndrome	The article discusses the development of a deep learning-based surrogate model for real-time diagnosis of Acute Respiratory Distress Syndrome (ARDS) using the Nottingham Physiology Simulator. The process involved using high-performance computing (HPC) to create lightweight models that efficiently simulate ARDS progression, achieving accuracy comparable to more complex systems. The project demonstrated the utility of HPC in rapidly developing and deploying diagnostic support tools in clinical settings.	Nottingham Physiology Simulator; MATLAB, Python, TensorFlow, Keras, Ray Tune	The Nottingham Physiology Simulator (NPS) is utilized alongside MATLAB and Python with libraries such as TensorFlow and Keras for deep learning tasks, and Ray Tune for hyperparameter tuning within an HPC environment to develop and test the model.	Not specified	Not mentioned	Intensive Care Unit (ICU)	The surrogate model developed mimics the performance of the original NPS, showing high predictive accuracy and enabling faster diagnosis, thus potentially improving treatment outcomes.	Not mentioned

GPT4o Responses

Article title	Summary	Software/AI used	Software/AI Description	Participants	Control and other groups	Hospital Area	Learning Impact	Student Satisfaction
Transformative Learning through Virtual Patient Simulations: Predicting Critical Student Reflections	This study explores the impact of virtual patient simulations on transformative learning in nursing education. Using the Digital Clinical Experience (DCE) from Shadow Health, the study analyzes self-reflections of 130 Master of Science in Nursing (MSN) students. The results show that critical self-reflection, indicating transformative learning, is predicted by the number of secondary clinical items discovered by students during their virtual patient interviews. The study found that deeper exploration of secondary clinical items led to higher levels of critical reflection.	Digital Clinical Experience (DCE)	A web-based virtual patient simulation software that allows nursing students to interview, examine, document, and reflect on virtual patient interactions. It includes various modules for different health assessments and provides structured self-reflection prompts.	130 MSN students	No specific control group; study focused on different levels of clinical findings discovered by students.	Nursing	- Encourages deep, critical self-reflection - Promotes clinical reasoning skills - Enhances ability to apply knowledge to different contexts - Improved clinical reasoning skills - Greater engagement in self-assessment - Enhances clinical reasoning and application to practice	Not specifically mentioned, but implications for practice suggest positive impacts on student engagement and learning.
Hospital nurses' management of agitation in older cognitively impaired patients: do they recognise pain-related agitation?	This study investigates how hospital nurses manage agitation in older cognitively impaired patients experiencing pain. Using virtual simulation, the study aimed to identify the gap in nurses' recognition and treatment of pain-related agitation. The results indicated that a majority of nurses administered antipsychotics instead of treating pain, with only 4.7% correctly identifying and managing the agitation as pain-related.	Virtual Human Toolkit	A suite of modules and tools for creating interactive virtual human conversational agents developed by the Institute of Creative Technologies at the University of Southern California. The simulation included branching pathways, video scenarios, and an avatar to mimic real-life interactions.	274 registered medical and surgical nurses from 10 public hospitals in Queensland, Australia.	No specific control group. The study involved different groups of nurses based on demographics, seniority, workplace, training, experience, and gerontology-specific knowledge.	Medicine, Nursing (medical and surgical settings), Medical and surgical units	1. Identification of a significant gap in recognizing and managing pain-related agitation.<br>2. Highlighting the need for better training and awareness among nurses to address pain as a potential cause of agitation.<br>- Revealed a gap in practice regarding pain recognition.<br>- Identified reliance on antipsychotics.	Not explicitly mentioned. The study focused more on the effectiveness of simulation and the gap in clinical practice.
User Response to the Simulation of a Virtual Patient with Cranial Nerve Injury	The study evaluates the Neurological Examination Rehearsal Virtual Environment (NERVE) tool, which simulates a virtual patient with cranial nerve injury using speech recognition and a Nintendo Wiimote®. It assesses user reception, ability to identify cranial nerve lesions, and overall satisfaction among medical students, residents, and clinicians.	NERVE, Nintendo Wiimote®	NERVE simulates a life-size virtual patient for cranial nerve exams, utilizing speech recognition and motion tracking with a Nintendo Wiimote® as a virtual hand, ophthalmoscope, and eye-chart.	69 participants: 9 clinicians, 7 residents, 8 MS3 and 4s, 20 MS2s, and 25 MS1s	No control group. Groups include clinicians, residents, and medical students across different years	Medicine	- Identified cranial nerve deficits<br>- Practiced history taking and examination skills<br>- Residents showed the most significant need for repeat exposure	Overall positive reception, residents found it most resembling an actual patient experience despite having the lowest correct identification rates
Developing a Conversational Virtual Standardized Patient to Enable Students to Practice History-Taking Skills	This study describes the development and evaluation of a virtual standardized patient (VSP) system where medical students practice history-taking and diagnostic skills using natural language interactions. The VSPs are created using ChatScript for dialogue management and Unity for 3D character simulation. The study found that VSPs effectively helped students practice and improve their history-taking skills, with high accuracy in student-VSP interactions (79%-86%). Future improvements include automatic assessment modules for immediate feedback.	ChatScript, Unity	ChatScript: an NLP engine for managing doctor-patient dialogues. Unity: a high-fidelity game engine for creating 3D characters.	52 students (18 third-year and 34 first-year)	No specific control group mentioned. Groups: Third-year and first-year students were tested separately with different case complexities.	General Internal Medicine, Family Medicine, Emergency Medicine	- Improved accuracy in history-taking skills.<br>- Ability to develop appropriate differential diagnoses.<br>- Enhanced practice before real patient interactions.<br>- Enhanced learning of clinical decision-making skills.<br>- Enhanced documentation skills in an EMR.	Not specifically mentioned
Affect and Cognitive Closure in Students—A Step to Personalised Education of Clinical Assessment in Psychology with the Use of Simulated and Virtual Patients	The study investigates the impact of affect and cognitive closure on learning performance using simulated patients (SP) and virtual patients (VP) among 56 fifth-year clinical psychology students. The study aims to measure the effectiveness of SP and VP in teaching psychological interview techniques and clinical reasoning. The results indicate that SP was more effective for interview skills, while VP was beneficial for most clinical reasoning skills. The findings suggest that affect and cognitive closure can be used to tailor personalized education for psychology students.	IBM SPSS 25, VP computer program, SP tools	IBM SPSS 25: Statistical analysis software used for data analysis; VP: A computer-based tool that assesses clinical interview skills through short recordings of diagnostic psychological consultations, providing feedback and assessing students' clinical reasoning skills; SP: Role-playing by trained individuals to teach and measure psychological interview	56 fifth-year clinical psychology students	SP group (25 initial, 24 final), VP group (31 initial, 27 final)	Clinical psychology, Psychology	- SP improved interview skills.<br>- VP enhanced clinical reasoning skills.<br>- SP beneficial for high EFNC students.<br>- VP beneficial for low EFNC students.<br>- VP provided structure for students with low EFNC, while SP helped those with high EFNC build on structuring skills.	Yes, detailed analysis showed positive feedback on both SP and VP methods, with specific preferences based on EFNC levels. Not explicitly mentioned.
Using a Virtual Patient via an Artificial Intelligence Chatbot to Develop Dental Students' Diagnostic Skills	The study evaluates the use of an AI chatbot, named Julia, as a virtual patient to help dental students at the Universidad Europea de Madrid improve their diagnostic skills for pulp pathology. The study involved fourth and fifth-year dental students who interacted with Julia via Telegram over several weeks and then completed a survey. Results showed high satisfaction among students, particularly those in their fifth year, and those who made a correct diagnosis rated the technology more positively.	Dialogflow	Dialogflow® is an intuitive AI application used to create conversational chatbot flows capable of understanding human language nuances through natural language processing and machine learning.	193 students (58 fourth-year, 135 fifth-year) (109 females, 84 males)	No explicit control group mentioned. Groups compared by year (fourth vs. fifth-year) and sex.	Dentistry	- Improved communication skills - Increased confidence and security - Enhanced professionalism - Effective in completing diagnostic questions - Positive evaluation of AI integration - High recommendation for inclusion in the curriculum	Yes, high satisfaction reported, particularly by fifth-year students, with a mean satisfaction score of 4.36.
Learning clinical reasoning with virtual patients	The article discusses the development and implementation of a virtual patient (VP) system utilizing AI and natural language processing to enhance clinical reasoning skills in medical students. The VP system allows students to interact with a simulated patient in real-time, making diagnoses and receiving instant feedback. The study involved 112 medical students from Kaohsiung Medical University and E-Da Hospital in Taiwan. The findings indicated high appreciation among students, with reported increases in confidence, proficiency, and organized thought processes.	AI and Natural Language Processing	A virtual patient with AI and natural language processing capabilities that recognizes and responds to students' verbal questions, providing real-time feedback and modifying responses based on students' decisions. It displays expert-derived schemata and generates performance scores with feedback.	112 medical students	Not specified; field testing only involved medical students.	Medicine and Pediatrics	- Early clinical exposure<br>- Repetitive practice with feedback<br>- Improved knowledge structure<br>- Increased confidence and proficiency in real-life patient scenarios	Yes, up to 80% of students reported increased confidence and proficiency. High satisfaction reported with students finding the learning experience interesting and expressing a desire for more VP-based simulations.
The virtual patient as a learning tool: a mixed quantitative qualitative study	This study evaluates the effectiveness of a low-fidelity virtual patient (VP) simulator in developing clinical reasoning and history-taking skills among undergraduate medical students. A course using the VP as the primary teaching tool was conducted, and student performance was assessed through a mix of quantitative and qualitative methods before and after the course. Significant improvements were observed in all evaluated aspects.	Virtual Patient (VP)	A web-based tool that simulates real clinical scenarios, allowing students to interact through typed questions and access physical exam findings. Students to interact through typed questions and access physical exam findings.	20 undergraduate medical students	No control group mentioned. The study involved the same group of students evaluated before and after the intervention.	Medicine	- Significant improvement in interview skills, physical exams, clinical judgment, relevance of medical exams, and case presentation. - Enhanced logical and structured thinking processes. - Increased ability to review and reassess information. - Significant improvement in history taking, clinical reasoning, physical exams, relevance of medical exams, and case presentation. - Encouraged logical and structured processes. - Allowed practice without consequences and enabled review and reassessment of [illegible]	Yes. Students found the VP to be an easy-to-use, motivating, and stress-free learning tool, especially useful at the beginning of their careers. They appreciated the ability to make mistakes without real consequences. Students found the VP motivating and easy to use, especially early in their careers. High; students found the VP tool easy to use, motivating, and helpful in creating logical and structured processes without the stress of real patient interaction.
Evaluation of an interactive virtual patient system (ISP) in an OSCE clinical assessment	This study evaluates the use of an Interactive Simulation of Patients (ISP) in an OSCE exam with 118 fourth-year medical students across four teaching hospitals. The ISP was integrated as one of the twelve examination modules to assess clinical reasoning and problem-solving abilities. Results showed that the ISP could differentiate student performance, though the presence of assistants affected outcomes. Students showed enhanced clinical reasoning skills and found the system engaging and realistic.	Interactive Simulation of Patients (ISP)	A comprehensive, high-fidelity virtual patient-based learning tool designed to help medical students practice clinical reasoning skills. It includes functions for medical history taking, physical exams, lab tests, and diagnosis suggestions in unscripted, authentic interactions.	118 students (110 participated; 8 excused)	Students were divided into subgroups at four hospitals (H1, H2, H3, H4) and rotated through different stations, including ISP and standardized patients.	Medicine (surgery course)	- Differentiated student performance effectively - Enhanced clinical reasoning skills - Better engagement with realistic scenarios	- 63% found the system engaging - 78% found it realistic - Mixed; overall opinions of the surgery course and OSCE exam rated higher than the ISP program. Some hospitals rated ISP lower due to first-time use and quality of assistance
A Study to Design a Learning Tool "Virtual Patient" for Functional Diagnosis and Clinical Reasoning of Respiratory Dysfunction in the Undergraduate Physiotherapy Curriculum	The study aimed to develop a software-based "virtual patient" for teaching functional diagnosis and clinical reasoning of respiratory dysfunction in undergraduate physiotherapy students. The study included creating a virtual environment using Oculus Quest, assessed its effectiveness through feedback from students and faculty, and found it well-organized and effective as a teaching tool.	Oculus Quest, Blender, IBM Watson Model, Unity	Oculus Quest for VR platform, Blender for modeling and animation, IBM Watson Model for text-to-speech interaction, and Unity for assembling the virtual environment	98 students and 15 faculty members	No specific control group; feedback collection from students and faculty	Physiotherapy	Improved clinical reasoning, diagnostic skills, analytical thinking, communication skills, and decision-making skills. Facilitated analytical thinking, problem-solving skills, and reduced anxiety.	High satisfaction; engaging, interesting, convenient, and supportive of curriculum inclusion. Positive feedback on clinical postings, skills development, and understanding enhancement.
Learning and clinical reasoning experience of second-year medical pharmacology students and teachers with virtual patients developed using OpenLabyrinth	The study evaluated the use of virtual case scenarios developed using OpenLabyrinth among second-year medical students and pharmacology teachers. Feedback indicated high perceived authenticity and relevance to real-life medical decision-making. The software allowed for branched decision-making paths in clinical case scenarios, enhancing the learning experience. The study concluded that virtual patient scenarios are well-received and useful in medical education, particularly in resource-limited settings.	OpenLabyrinth	OpenLabyrinth is a server-based software platform that enables the creation and use of interactive virtual patient scenarios. It allows for remote access and includes tools for incorporating multimedia elements and branched decision-making paths, providing a risk-free environment for medical training.	32 (20 students, 12 teachers)	No control group; only participating students and teachers	Pharmacology	1. Improved understanding of patient interaction<br>2. Enhanced decision-making skills<br>3. Better preparedness for real-life scenarios<br>4. Enhanced interprofessional collaboration<br>5. Provided a broader perspective on patient care<br>6. Encouraged proper communication and ethical behavior	Yes, positive feedback from both students and teachers. 95% of students and all faculty felt the virtual scenarios were realistic and educational.
